# Traumatic events, other operational stressors and physical and mental health reported by Australian Defence Force personnel following peacekeeping and war-like deployments

**DOI:** 10.1186/1471-244X-12-88

**Published:** 2012-07-26

**Authors:** Michael Waller, Susan A Treloar, Malcolm R Sim, Alexander C McFarlane, Annabel C L McGuire, Jonathan Bleier, Annette J Dobson

**Affiliations:** 1 The University of Queensland, Centre for Military and Veterans Health, Mayne Medical School, University of Queensland, Herston, QLD, 4006, Australia; 2 Monash Centre for Occupational and Environmental Health Department of Epidemiology & Preventive Medicine, Medical School, Monash University Alfred Hospital, Melbourne, VIC, Australia; 3 Centre for Traumatic Stress Studies, The University of Adelaide, 122 Frome Street, Adelaide, South Australia, Australia; 4 Medical Research Council Clinical Trials Unit, London, UK

## Abstract

**Background:**

The association between stressful events on warlike deployments and subsequent mental health problems has been established. Less is known about the effects of stressful events on peacekeeping deployments.

**Methods:**

Two cross sectional studies of the Australian Defence Force were used to contrast the prevalence of exposures reported by a group deployed on a peacekeeping operation (Bougainville, n = 1704) and those reported by a group deployed on operations which included warlike and non-warlike exposures (East Timor, n = 1333). A principal components analysis was used to identify groupings of non-traumatic exposures on deployment. Multiple regression models were used to assess the association between self-reported objective and subjective exposures, stressors on deployment and subsequent physical and mental health outcomes.

**Results:**

The principal components analysis produced four groups of non-traumatic stressors which were consistent between the peacekeeping and more warlike deployments. These were labelled ‘separation’, ‘different culture’, ‘other people’ and ‘work frustration’. Higher levels of traumatic and non-traumatic exposures were reported by veterans of East Timor compared to Bougainville. Higher levels of subjective traumatic exposures were associated with increased rates of PTSD in East Timor veterans and more physical and psychological health symptoms in both deployed groups. In Bougainville and East Timor veterans some non-traumatic deployment stressors were also associated with worse health outcomes.

**Conclusion:**

Strategies to best prepare, identify and treat those exposed to traumatic events and other stressors on deployment should be considered for Defence personnel deployed on both warlike and peacekeeping operations.

## Background

People exposed to stressful events on deployment are more likely to report subsequent mental health problems, in particular symptoms of Post Traumatic Stress Disorder (PTSD) or alcohol misuse [1-4]. This finding has been well documented in studies focused on combat exposures during deployments to Vietnam, Iraq and Afghanistan. Less is known about the level of traumatic exposure experienced on peacekeeping deployments and the effects of this type of exposure on subsequent health [5].

Many of the recent studies of peacekeeping deployments have focused on operations in Somalia and Bosnia. A 12 % absolute increase in psychiatric morbidity was found in a small study of Australian troops who deployed to Somalia [6], and higher levels of anxiety, depression and psychological distress were found in a study of New Zealand peacekeepers [7] deployed predominantly to Cambodia. A study of UK personnel showed a twofold increase in heavy drinking in those who had deployed to Bosnia [8], but this study and others of deployments to this region have not shown an increase in psychiatric morbidity or PTSD in deployed troops [9,10]. However, the differing locations and exposures associated with the operations studied make it difficult to generalise these results to other peacekeeping deployments [5].

A number of stressors have been associated with peacekeeping operations, including environmental adaptation and homesickness [11], pre-deployment anxiety [7,9], and witnessing atrocities [6]. Each of these stressors has the potential to affect the physical and mental health of those deployed. Personnel deployed on peacekeeping missions may also be exposed to warlike stressors and potentially dangerous situations. One of the most common stressors (65 %-83 %) reported by UK and US troops deployed to Bosnia and Kosovo was danger of minefields [9,12]. Being shot at or coming under small arms fire (35 %-49 %) and witnessing human remains (40 %-53 %) [9,13] were also commonly reported. Likewise US peacekeepers deployed to Somalia frequently experienced dangerous patrols and having rocks thrown at their unit [14]. In the face of these exposures peacekeepers have noted uncertainty over their role and frustration in complying with the rules of engagement [6,9,12,14].

Previous research has shown increased rates of PTSD among those reporting events such as sustaining wounds or witnessing injuries or death [2,15], however, other studies have reported that fear of events on deployment are also strong predictors of PTSD [2,16-18]. Those who have experienced more traumatic exposures may have worse psychological health because repeated exposures may have ‘sensitized’ them to reactions to trauma related cues [19], however, repeated exposures may have ‘immunized’ others to such a reaction [13]. Non-traumatic deployment stressors have also been shown to be associated with worse health outcomes [1].

Recent years have seen a marked increase in Australia’s operational commitments abroad. Between 1980 and 1989 the Australian Defence Force (ADF) had 16 operational deployments involving a total of approximately 1,000 personnel. In contrast, nearly 17,000 were deployed between 1990 and 1999. Further, large deployments to East Timor (19,705 personnel deployed between June 1999 and May 2005) and Iraq and Afghanistan (26,915 personnel deployed between 2001 and December 2009) have occurred since then. These deployments are a significant commitment for the ADF, which employs approximately 50,000 permanent full-time active duty personnel. The ADF defines non-warlike operations as those where there is risk associated with the assigned tasks, where the application of force is limited to self-defence, and where casualties could occur but are not expected [20]. The deployment to Bougainville was classified as ‘non-warlike’ whereas the East Timor deployment included both ‘warlike’ and ‘non-warlike’ operations.

Against the backdrop of increasing operational tempo, the Deployment Health Surveillance Program was established to provide a systematic, prospective and ongoing means of assessing and understanding the health effects of operational deployment on ADF personnel. As part of this program, studies of the post deployment health of those deployed to Bougainville and East Timor and comparison groups have been undertaken.

A recent literature review of peacekeeping deployments highlighted the need for a comparison of soldiers undergoing deployment on combat operations and peacekeeping operations to delineate whether the stressors of peacekeeping deployments differ meaningfully from those in combat operations [5]. In this paper we assess how frequently peacekeepers report traumatic and non-traumatic stressors compared to those on a deployment including warlike and non-warlike operations.

For each deployment group we assess the association between the number of traumatic events experienced on deployment and self reported psychological and physical health outcomes. We also consider objective and subjective traumatic exposures separately to determine which are most strongly related to physical and psychological health outcomes. Finally, we will assess the relationship between non-traumatic deployment stressors and physical and mental health.

## Methods

### Study groups

The Bougainville and East Timor Health studies were cross-sectional studies undertaken in 2008. In this paper we focus on the deployed groups in each study. The Bougainville study invited the full nominal roll of 4,775 veterans. The sample for the East Timor study had a veteran group of 3,999 drawn from a nominal roll of 19,705 which was generated to be representative of the nominal roll based on Service, service type (permanent or reserve), age group and gender using stratified random sampling.

### Recruitment

An invitation was sent to all sampled individuals informing them about the study and participants could complete a paper or online questionnaire. Reminder cards/emails were sent within one month and follow-up phone calls were then made to non-responders. The period of recruitment for the study was November 2007 to January 2009. Informed consent for participation in the study was obtained from each participant.

These studies were approved by the Australian Defence Human Research Ethics Committee, the Department of Veterans’ Affairs Human Research Ethics Committee and the University of Queensland Behavioural & Social Sciences Ethical Review Committee.

### Characteristics of deployment

The dates and nature of the operations included in the Bougainville and East Timor studies are detailed in Table 1. ADF personnel were deployed to Bougainville as part of the New Zealand-led Truce Monitoring Group and the Australian-led Peace Monitoring Group.

**Table 1:** Dates of operations for East Timor and Bougainville studies

**Location**	**Operation**	**Type of Operation**	**Start date**	**End date**
***Bougainville***	BEL ISI I	Non-warlike	Nov 1997	Apr 1998
	BEL ISI II	Non-warlike	Apr 1998	Aug 2003
***East Timor***	FABER	Non-warlike	Jun 1999	Sep 1999
		Warlike	Sep 1999	Feb 2000
	SPITFIRE	Non-warlike	Sep 1999	Sep 1999
	WARDEN	Warlike	Sep 1999	Apr 2000
	TANAGER	Warlike	Feb 2000	May 2002
	CITADEL	Warlike	May 2002	Aug 2003
		Non-warlike	Aug 2003	May 2004
	SPIRE	Non-warlike	May 2004	May 2005

### Measurements

The Traumatic Stress Exposure Scale – Revised (TSES-R2) is designed to measure the frequency and severity of traumatic events during deployment [21]. The instrument was originally derived after a peacekeeping deployment to Rwanda and has been used extensively in the ADF in peacekeeping and combat settings. The first subscale of this instrument (TSES-R2-A) asks participants to record the frequency with which they experienced 12 separate events (e.g., “You were in danger of being killed” or “You had to handle dead bodies”). Two subsequent subscales (TSES-R2-B and TSES-R2-C) then ask “How did it affect you at the time?” and “How does it affect you now?”. For the purposes of this analysis the TSES-R2-A was divided into 7 objective (handled dead bodies, saw dead bodies, heard of a friend injured or killed, present when a friend injured or killed, witness to human degradation, heard of a loved one injured or killed, present when a loved one was injured or killed) and 5 subjective exposures (danger of being killed, danger of being injured, feared exposure to disease or toxic agent, believed actions or inaction resulted in injury, believed actions or inaction resulted in death) to assess the effect of each group of exposures separately. A score for the objective and subjective scales was generated by applying the following values to the responses; 0 = Never, 1 = Rarely (1 time), 2 = Occasionally (2–5 times), 3 = Often (6–10 times), 4 = Very Often (11+ times). For the purposes of assessing the prevalence of each exposure the items were dichotomised to ‘never’ or ‘ever’.

A list of 36 non-traumatic stressors on military operations was also used [22] to quantify the level of non-traumatic military stress experienced in Bougainville and East Timor. Participants were asked to rate each stressor on a 5 point scale from ‘no stress’ to ‘extreme stress’. This scale included items such as “separation from family and friends” and “behaviour of others”. This scale was developed in 1990s based on stressful experiences that had been raised by ADF members and was routinely used in psychological interviews to ‘prompt’ members to discuss stressful experiences. The internal consistency of this scale on our respondents was very good (Cronbach’s alpha =0.95).

Outcome measures were chosen to assess the effect of traumatic exposures on the mental health and the reporting of more general health symptoms. A 67 item symptom checklist used was adapted from the Australian Gulf War Veteran Health Study [23] and Op TELIC study of UK Gulf War veterans [24]. This instrument was based on the Hopkins Symptoms Checklist and included both physical (e.g., chest pain, headaches) and psychological symptoms (e.g., forgetfulness, loss of concentration). This checklist had good internal consistency among the study population used in this analysis (Cronbach’s alpha =0.95). Respondents were asked to grade the occurrence of each symptom in the previous four weeks as “None”, “Mild”, “Moderate” or “Severe”. The total number of these physical and psychological health symptoms was calculated by combining the number of mild, moderate and severe symptoms reported.

The Kessler Psychological Distress Scale (K10) was used to measure non-specific psychological distress. Cut-offs for this scale have been developed by the Clinical Research Unit for Anxiety and Depression, School of Psychiatry, University of New South Wales to determine the prevalence of anxiety and depressive disorders. Using these cut-offs, people who score above 29 on this scale were considered to have a high level of psychological distress [25]. Data from the Australian National Survey of Mental Health and Wellbeing (NSMHWB) and from our study of Bougainville and East Timor veterans showed this scale had good internal consistency (Cronbach’s alphas = 0.92 and 0.93 respectively).

The Post-Traumatic Stress Disorder Checklist Civilian Version (PCL-C) was also used [26]. This is a self-report rating scale for assessing 17 symptoms of PTSD [27]. A cut-off of 50 on the PCL-C has previously been used as an indicator of PTSD prevalence in military populations [28]. This scale has demonstrated high internal consistency from our study of Bougainville and East Timor veterans (Cronbach's alpha =0.95) and similar estimates between 0.94 [29] and 0.97 [26] in other studies.

### Statistical analysis

The frequency of reporting each traumatic and non-traumatic event at least once was compared between the Bougainville and East Timor veterans using the chi-squared test or Fisher’s exact test. No adjustment was made for people who deployed to both locations.

Principal components analyses were undertaken to reduce the 36 non-traumatic military stressors into smaller subgroups of variables so that the effect of different groupings of non-traumatic stress could be assessed. A varimax rotation was used, as the results would be used in subsequent regression analyses. Initially the data from East Timor and Bougainville were analysed separately but as they gave similar factor structures they were re-analysed together. The choice of factors was made based on the number of eigenvalues > 1, the number of eigenvalues that explained more than 60 % of the variance, an examination of the scree plot, and the interpretability of the results. Factor scores were calculated by creating a count of the number of non-traumatic stressors reported in each factor group for each participant.

The TSES-R2 scales and the counts of items from the factors of the non-traumatic stressors checklist had skewed distributions. Between-group (Bougainville v East Timor) and within-person comparisons (“How did it affect you then” v “How does it affect you now”) of these scores were made using the Wilcoxon-Mann–Whitney test and the Wilcoxon signed rank sum test respectively.

Health outcomes were compared between people who reported different levels of traumatic events and non-traumatic events based on the quartiles of the total scores for subjective and objective items on the TSES-R2-A, which accounted for the frequency of each event, and quartiles of the factor scores of the non-traumatic stressors. The non-traumatic factor scores were calculated by summing the non-traumatic stressors in each factor. The quartiles used were calculated based on the combined dataset of East Timor and Bougainville. These quartiles were also used to test for trend of increased exposure and health outcomes in regression models.

The total number of symptoms reported was compared across the TSES-R2-A quartiles using negative binomial regression which is appropriate for over-dispersed count data, and logistic regression was used to compare dichotomous health outcomes (K10 ≥ 30, PCL-C ≥ 50) between those who reported different levels of stressful events on deployment. All models were adjusted for age (20–29, 30–39, 40+ years), gender, Service (Navy, Army, RAAF) and rank (officer or enlisted) and factor scores for the non-traumatic military stressors derived from the principal components analyses. In the analysis of traumatic exposures (TSES-R2-A) and the number of symptoms reported, we also adjusted for the K10 score, to assess how the level of psychological distress was associated with the reporting of current physical symptoms.

In recent years the ADF has deployed to Afghanistan (from 2001) and Iraq (from 2003). Twenty-two percent of the Bougainville veterans and 26 % of the East Timor veterans had deployed to Iraq or Afghanistan. Adjusting for these deployments in the statistical models caused only a slight change in the estimates. The models presented in the Results section are not adjusted for deployments to the Middle East.

Statistical analyses were performed using SAS version 9.2 [30] and STATA version 10.1 [31].

## Results

In both studies the median time since date of first deployment to the country and start of the study was eight years. The response rates for the deployed groups in the Bougainville and East Timor studies were 49 % (n = 2342) and 46 % (n = 1825) respectively, but comparisons of health outcomes are limited to those who completed the TSES-R2-A (n = 1704 and n = 1333). The demographic characteristics of the study participants who did and did not complete the TSES-R2-A are detailed in Table 2. In both studies response rates were higher in older age groups, officers and those still serving in the ADF (Table 2). Males and females did deploy to East Timor and Bougainville, however the overall ratio of females to males was low (Table 2). Accordingly, separate analyses for female ADF members have not been included in this paper but adjustments for gender have been made in the analyses. Of the responders 136 personnel were in both Bougainville and East Timor datasets.

**Table 2:** Demographic characteristics of Bougainville and East Timor participants

**Characteristic**		**Bougainville veterans who responded to TSES-R2 n (%)**	**Bougainville veterans who did not respond to TSES-R2 n (%)**	**Bougainville veterans who did not respond to Questionnaire n (%)**	**East Timor veterans who responded to TSES-R2 n (%)**	**East Timor veterans who did not respond to TSES-R2 n (%)**	**East Timor veterans who did not respond to Questionnaire n (%)**
Gender	Male	1465 (86)	566 (89)	2084 (86)	1184 (89)	440 (88)	2006 (93)
	Female	239 (14)	72 (11)	349 (14)	149 (11)	60 (12)	160 (7)
Age group	20-29	112 (7)	67 (11)	365 (15)	178 (13)	75 (15)	570 (26)
	30-39	756 (44)	311 (49)	1266 (52)	648 (49)	269 (54)	1093 (50)
	40+	836 (49)	260 (41)	802 (33)	507 (38)	156 (31)	503 (23)
Service	Navy	382 (22)	203 (32)	846 (35)	176 (13)	63 (13)	218 (10)
	Army	1245 (73)	404 (63)	1485 (61)	1025 (77)	389 (78)	1778 (82)
	RAAF	77 (5)	31 (5)	102 (4)	132 (10)	48 (10)	170 (8)
Rank	Officer	602 (35)	188 (29)	590 (24)	344 (26)	128 (26)	351 (16)
	Enlisted	1102 (65)	450 (71)	1834 (76)	989 (74)	372 (74)	1813 (84)
Status	Currently serving	1464 (86)	570 (89)	1612 (67)	1158 (87)	456 (91)	1488 (69)
	Ex-serving	240 (14)	68 (11)	812 (33)	175 (13)	44 (9)	678 (31)

In those who deployed to East Timor 7.2 % had symptoms of PTSD (PCL-C ≥50) and 6.9 % had a high level of psychological distress (K10 ≥30). The corresponding numbers in the Bougainville group were 5.9 % and 5.5 % respectively.

East Timor veterans reported significantly more traumatic events than those deployed to Bougainville (median TSES-R2-A 6 v 3, p < 0.0001) with the exception of ‘fearing exposure to contagious disease, toxic agents or injury’ which was reported at least once by 38 % of those deployed to Bougainville compared to 31 % in East Timor veterans (Table 3). The other common traumatic stressors recorded in both deployed groups were fear of injury, fear of death, witnessing dead bodies and seeing human degradation. Both groups reported that they were less affected by stressful experiences on deployment now, than they were at the time of the event (medians TSES-R2-B v TSES-R2-C: Bougainville 2 v 0, East Timor 3 v 0 p-values <0.0001).

**Table 3:** Traumatic exposures reported by Bougainville and East Timor Veterans

**Traumatic exposure**^**B**^	**Bougainville (n = 1793)**	**East Timor (n = 1432)**	
	**Prevalence**	**Prevalence**	
	**n (%)**	**n (%)**	**p- value**^**A**^
Danger of being killed	954 (53)	1022 (71)	<0.001
Danger of being injured	1028 (58)	1024 (72)	<0.001
Handled dead bodies	289 (16)	404 (28)	0.02
Saw dead bodies	472 (27)	689 (49)	<0.001
Heard of a close friend or co-worker, injured or killed	353 (20)	430 (30)	0.35
Present when a close friend or co-worker, injured or killed	164 (9)	184 (13)	1
You feared that you had been exposed to a contagious disease, toxic agent or injury	672 (38)	430 (31)	0.003
You were witness to human degradation and misery on a large scale	454 (26)	818 (58)	<0.001
You heard of a loved one injured or killed	189 (11)	178 (13)	0.13
You were present when a loved one was injured or killed	40 (2)	36 (3)	0.44
You believe your actions or inaction resulted in someone being seriously injured	72 (4)	103 (7)	0.81
You believe your actions or inaction resulted in someone being killed	50 (3)	74 (5)	0.27

^A^Chi-squared or Fisher’s exact test comparing the proportions exposed.

In both deployed groups some of most frequent non-traumatic stressors reported were separation from family and friends, sorting out problems at home, threat of danger, risk of motor vehicle accidents and the behavior of others. A four factor solution was selected to summarise the data from the principal components analysis of the non-traumatic military stressors (Table 4). The four factors were labeled ‘separation’, ‘different culture’, ‘other people’ and ‘work frustration’. Two items, ‘Taking leave in countries other than Australia’ and ‘Risk of unauthorised discharge of weapon’ were removed from subsequent analyses because they did not consistently load on any factor or they were reported with a low prevalence on both deployments (Table 4). When the total score of the items which made up the four factors was compared between East Timor and Bougainville veterans, those deployed to East Timor reported a higher level of non-traumatic stress (p-values < 0.001). Likewise 33 out of 36 non-traumatic stressors were reported more frequently in those deployed to East Timor than Bougainville veterans (Table 4).

**Table 4:** Non traumatic stressors on reported by those deployed to Bougainville and East Timor

**Non traumatic stressor**	**Bougainville (n = 1757)**	**Median (IQR)**	**East Timor (n = 1401)**	**Median (IQR)**^**B**^	**p-value**^**A**^
	**Prevalence**		**Prevalence**		
	**n (%)**		**n (%)**		
Factor 1 - Separation		12 (7)		13 (6)	<0.001
Contact with family/friends	957 (55)		777 (56)		0.54
Separation from family/friends	1168 (67)		980 (70)		0.04
Thinking about returning home	781 (45)		707 (51)		<0.001
Isolation from Australia	972 (56)		783 (56)		0.80
Sorting out problems at home	1037 (59)		892 (64)		0.008
Mail service	565 (32)		416 (30)		0.14
Length of deployment	469 (27)		536 (38)		<0.001
Taking leave back in Australia	288 (17)		373 (27)		<0.001
Factor 2 – Different culture		13 (6)		15 (7)	<0.001
Working with military of other cultures	356 (20)		500 (36)		<0.001
Threat of danger	998 (57)		933 (67)		0.006
Living in a different culture	539 (31)		482 (35)		0.03
Language barriers	529 (30)		539 (39)		<0.001
Completing deployment’s objectives	527 (30)		520 (37)		<0.001
Your role in the country	451 (26)		438 (31)		<0.001
Risk of vehicle accidents	1004 (57)		990 (71)		<0.001
Isolation from other deployed members	297 (17)		298 (21)		0.002
The overseas organisation	398 (23)		620 (45)		<0.001
Health concerns	799 (46)		721 (52)		<0.001
Taking leave in countries other than Australia	110 (6)		110 (8)		0.06
Factor 3 – Other people		17 (8)		18 (9)	<0.001
Living and working with the same people	821 (47)		713 (51)		0.02
Not getting on with others	616 (35)		559 (40)		0.006
Behaviour of others	1046 (60)		971 (70)		<0.001

^A^Chi-squared comparing the proportions exposed or signed rank test to compare median count of stressors in each factor group.

^B^Score of symptoms in each factor group.

Among Bougainville veterans there was no significant association between the level of subjective or objective traumatic exposures and reporting high psychological distress (K10 ≥ 30) (Table 5). Bougainville veterans who reported 1, or more than 3 subjective stressors were more likely to also report PTSD. However, the test for trend between subjective stressors and PTSD was not significant (p = 0.08, Table 5). Those who reported more subjective traumatic exposures also reported more items on the symptoms checklist (test for trend p = 0.001, Table 5). Some of the non-traumatic stressors were associated with health outcomes in those deployed to Bougainville. In particular those who reported more ‘work frustration’ stressors were more likely to report PTSD (test for trend p = 0.04, Table 5) and more items on the symptoms checklist (test for trend p = 0.01, Table 5). ‘Other people’ and ‘Cultural’ stressors were also associated with increased reporting of PTSD (tests for trends p = 0.02, p = 0.03, Table 5).

**Table 5:** Non-traumatic and traumatic experiences in Bougainville Veterans and subsequent physical and psychological health outcomes (n=1704)

	**K10**		**PCL-C**		**Symptoms**	
	**K10>29 n (%)**	**Odds Ratio 95% CI**^**A**^	**PCL-C>49 n (%)**	**Odds Ratio 95% CI**^**A**^	**Mean (SD)**	**Ratio of means 95% CI**^**B**^
**Objective stressors**						
0	38 (4.8)	1 (Reference)	28 (3.6)	1 (Reference)	13.0 (10.6)	1 (Reference)
1	17 (6.4)	1.06 (0.55, 2.03)	16 (6.5)	1.15 (0.55, 2.42)	14.2 (11.1)	0.96 (0.87, 1.06)
2-3	21 (6.4)	0.98 (0.52, 1.85)	24 (7.6)	1.31 (0.67, 2.57)	15.6 (12.6)	0.98 (0.89, 1.07)
4+	20 (7.1)	0.91 (0.46, 1.80)	24 (8.9)	1.06 (0.52, 2.15)	18.1 (13.1)	1.05 (0.94, 1.17)
Test for trend p-value		0.79		0.76		0.60
**Subjective stressors**						
0	24 (4.4)	1 (Reference)	12 (2.3)	1 (Reference)	11.1 (9.9)	1 (Reference)
1	9 (5.4)	1.10 (0.45, 2.71)	12 (7.5)	2.91 (1.08, 7.85)	13.4 (11.1)	1.08 (0.95, 1.24)
2-3	22 (4.8)	0.89 (0.44, 1.77)	16 (3.6)	1.07 (0.43, 2.68)	14.3 (10.5)	1.09 (0.99, 1.21)
4+	44 (8.6)	1.17 (0.58, 2.36)	55 (11.1)	2.51 (1.06, 5.95)	19.0 (13.2)	1.20 (1.08, 1.33)
Test for trend p-value		0.75		0.08		0.001
**Separation stressors**						
8	11 (3.5)	1 (Reference)	6 (2.0)	1 (Reference)	9.6 (9.3)	1 (Reference)
9-11	17 (4.0)	0.75 (0.32, 1.78)	9 (2.2)	0.47 (0.15, 1.49)	11.9 (9.9)	1.04 (0.93, 1.17)
12-15	18 (3.8)	0.42 (0.16, 1.07)	20 (4.3)	0.42 (0.13, 1.31)	14.8 (11.0)	1.09 (0.97, 1.23)
16-40	53 (12.5)	1.01 (0.39, 2.57)	56 (13.8)	0.66 (0.21, 2.10)	20.7 (12.9)	1.07 (0.94, 1.23)
Test for trend p-value		0.60		0.72		0.27
**Culture stressors**						
10	11 (3.5)	1 (Reference)	3 (1.0)	1 (Reference)	8.0 (7.6)	1 (Reference)
11-13	22 (3.9)	0.76 (0.32, 1.79)	14 (2.6)	1.58 (0.38, 6.48)	12.9 (9.9)	1.29 (1.15, 1.45)
14-17	20 (4.8)	0.65 (0.24, 1.75)	21 (5.3)	1.97 (0.44, 8.83)	15.9 (10.9)	1.32 (1.15, 1.52)
18-50	44 (12.8)	1.20 (0.40, 3.57)	56 (16.8)	3.71 (0.78, 17.7)	22.3 (13.5)	1.44 (1.22, 1.70)
Test for trend p-value		0.48		0.03		<0.001
**Other people stressors**						
11-12	7 (2.1)	1 (Reference)	3 (0.9)	1 (Reference)	8.6 (7.9)	1 (Reference)
13-16	18 (3.9)	2.26 (0.79, 6.44)	11 (2.4)	1.70 (0.37, 7.83)	12.0 (9.5)	1.09 (0.97, 1.23)
17-21	27 (6.0)	2.80 (0.85, 9.26)	21 (4.8)	2.14 (0.41, 11.17)	15.4 (10.5)	1.10 (0.96, 1.26)
22-55	47 (11.9)	3.54 (0.97, 12.90)	59 (15.5)	4.31 (0.78, 23.83)	22.0 (13.7)	1.14 (0.96, 1.34)
Test for trend p-value		0.09		0.02		0.18
**Work frustration stressors**						
5	15 (2.9)	1 (Reference)	7 (1.4)	1 (Reference)	9.7 (8.7)	1 (Reference)
6-7	17 (4.4)	1.16 (0.51, 2.67)	13 (3.4)	1.32 (0.42, 4.15)	13.1 (10.2)	1.08 (0.97, 1.20)
8-10	14 (3.7)	0.68 (0.26, 1.72)	14 (3.8)	0.91 (0.27, 2.99)	15.6 (10.6)	1.15 (1.03, 1.29)
11-25	55 (13.9)	2.04 (0.83, 4.98)	61 (16.1)	2.38 (0.75, 7.56)	21.9 (13.7)	1.17 (1.03, 1.33)
Test for trend p-value		0.08		0.04		0.01

^A^ adjusted for age (20-29, 30-39, 40+), Sex, Service, Rank (officer or enlisted) and quartiles of traumatic and non-traumatic stress.

^B^ adjusted for age (20-29, 30-39, 40+), Sex, Service, Rank (officer or enlisted), K10 and quartiles of traumatic and non-traumatic stress.

In the East Timor veterans there was no significant association between the level of subjective or objective traumatic exposures and reporting high psychological distress (K10 ≥ 30), however, those who reported more subjective stressors on deployment were more likely to score above 49 on the PCL-C scale (test for trend p = 0.008, Table 6) and more items on the symptoms checklist (test for trend p < 0.001, Table 6). Each of the groups of non-traumatic stressors was significant in at least one test for trend with the outcomes. Increased reporting of ’work frustration’ stressors was associated with an increased level of PTSD, high psychological distress and more items on the symptoms checklist (Table 6) and ‘cultural stressors’ were also associated with increased reporting of PTSD (test for trend p = 0.03, Table 6).

**Table 6:** Non-traumatic and traumatic experiences in East Timor Veterans and subsequent physical and psychological health outcomes (n=1333)

	**K10**		**PCL-C**		**Symptoms**	
	**K10>29 n (%)**	**Odds Ratio 95% CI**^**A**^	**PCL-C>49 n (%)**	**Odds Ratio 95% CI**^**A**^	**Mean (SD)**	**Ratio of means 95% CI**^**B**^
**Objective stressors**						
0	16 (5.2)	1 (Reference)	13 (4.5)	1 (Reference)	11.9 (10.8)	1 (Reference)
1	7 (3.7)	0.46 (0.16, 1.37)	5 (2.7)	0.47 (0.14, 1.62)	12.4 (10.1)	1.01 (0.89, 1.16)
2-3	14 (4.1)	0.49 (0.21, 1.17)	13 (3.8)	0.41 (0.15, 1.12)	15.0 (11.3)	1.06 (0.95, 1.20)
4+	59 (12.1)	1,05 (0.50, 2.24)	67 (14.1)	1.35 (0.58, 3.11)	18.6 (13.5)	1.05 (0.94, 1.19)
Test for trend p-value		0.49		0.12		0.33
**Subjective stressors**						
0	12 (4.7)	1 (Reference)	6 (2.5)	1 (Reference)	10.0 (9.6)	1 (Reference)
1	3 (2.4)	0.12 (0.02, 1.00)	2 (1.7)	-	12.5 (10.0)	1.29 (1.10, 1.51)
2-3	17 (4.4)	0.54 (0.22, 1.30)	16 (4.3)	1.31 (0.44, 3.88)	14.0 (10.1)	1.29 (1.14, 1.46)
4+	62 (10.9)	0.99 (0.43, 2.30)	72 (13.1)	2.48 (0.85, 7.23)	19.1 (13.3)	1.39 (1.23, 1.58)
Test for trend p-value		0.41		0.008		<0.001
**Separation stressors**^B^						
8	7 (3.8)	1 (Reference)	5 (2.9)	1 (Reference)	10.4 (10.1)	1 (Reference)
9-11	12 (3.4)	0.64 (0.22, 1.86)	14 (4.0)	0.99 (0.30, 3.26)	11.3 (9.8)	1.04 (0.91, 1.19)
12-15	21 (5.6)	0.69 (0.24, 2.01)	13 (3.6)	0.39 (0.11, 1.41)	15.0 (10.9)	1.14 (0.98, 1.31)
16-40	53 (12.9)	0.88 (0.29, 2.64)	64 (16.1)	0.81 (0.22, 2.90)	21.0 (13.3)	1.17 (1.00, 1.37)
Test for trend p-value		0.77		0.99		0.02
**Culture stressors**^B^						
10	3 (2.4)	1 (Reference)	5 (4.3)	1 (Reference)	8.7 (9.8)	1 (Reference)
11-13	15 (3.7)	1.90 (0.38, 9.49)	8 (2.1)	0.32 (0.07, 1.36)	11.1 (8.9)	1.03 (0.88, 1.21)
14-17	19 (5.2)	2.65 (0.49, 14.37)	14 (3.9)	0.66 (0.15, 2.87)	14.1 (10.4)	1.02 (0.86, 1.22)
18-50	62 (13.8)	3.91 (0.68, 22.61)	74 (17.0)	1.42 (0.31, 6.48)	21.7 (13.5)	1.05 (0.86, 1.28)
Test for trend p-value		0.06		0.03		0.65
**Other people stressors**^B^						
11-12	7 (3.5)	1 (Reference)	4 (2.1)	1 (Reference)	8.7 (8.8)	1 (Reference)
13-16	15 (4.3)	0.99 (0.31, 3.18)	13 (3.8)	1.62 (0.37, 7.01)	11.6 (9.8)	1.29 (1.10, 1.51)
17-21	16 (4.6)	0.83 (0.23, 3.01)	14 (4.2)	1.43 (0.28, 7.32)	15.1 (10.3)	1.29 (1.14, 1.43)
22-55	57 (12.9)	1.20 (0.32, 4.51)	68 (16.0)	2.04 (0.39, 10.67)	21.3 (13.5)	1.28 (1.08, 1.52)
Test for trend p-value		0.59		0.38		0.003
**Work frustration stressors**^B^						
5	8 (2.9)	1 (Reference)	8 (3.0)	1 (Reference)	9.6 (9.5)	1 (Reference)
6-7	7 (2.7)	1.16 (0.36, 3.72)	5 (2.0)	0.70 (0.19, 2.57)	12.0 (9.4)	1.08 (0.95, 1.23)
8-10	16 (4.7)	1.34 (0.45, 4.01)	11 (3.3)	0.71 (0.22, 2.35)	14.5 (10.6)	1.13 (0.99, 1.28)
11-25	67 (14.3)	2.79 (0.96, 8.12)	76 (16.9)	2.63 (0.88, 7.88)	21.2 (13.5)	1.15 (1.00, 1.32)
Test for trend p-value		0.02		0.004		0.05

^A^ adjusted for age (20-29, 30-39, 40+), Sex, Service, Rank (officer or enlisted) and quartiles of traumatic and non-traumatic stress.

^B^ adjusted for age (20-29, 30-39, 40+), Sex, Service, Rank (officer or enlisted), K10 and quartiles of traumatic and non-traumatic stress.

## Discussion

In Bougainville and East Timor veterans, subjective traumatic stressors, were a stronger predictor of PTSD and more physical and psychological symptoms than the objective traumatic stressors. Indeed the objective traumatic stressors considered were not predictive of any the health outcomes after adjustment for other traumatic and deployment stressors. In both deployed groups non-traumatic stressors were associated with negative health outcomes. However, other than the ‘work frustration’ stressors, which was associated with worse health outcomes in both Bougainville and East Timor groups, the relationship between other groups of non-traumatic stressors and health outcomes varied by deployment group and health outcome.

Consistent with the ‘warlike’ classification of a number of the East Timor operations, 11 of the 12 traumatic exposures were endorsed more frequently than the Bougainville veterans. The traumatic exposures with the highest prevalence in the East Timor group were ‘fear of death or injury’, ‘witnessing dead bodies’ and ‘human degradation’. Overall levels of traumatic exposures on deployment in both the Bougainville and East Timor groups were lower than those reported in recent studies of US personnel deployed to the Middle East. For example, in a study of US Marines the proportion who felt in danger of being killed ‘often or very often’ was 43 % [2], and in another analysis of US Marines, 77 % reported feeling they could have been killed at any time on at least one occasion [1]. In a study of US Army personnel, the prevalence of seeing dead bodies in Afghanistan (39 %) was below that in our study of East Timor veterans (49 %) but the prevalence reported by those deployed to Iraq was considerably higher (95 %) [15].

Groupings of non-traumatic stressors reported by East Timor and Bougainville veterans were similar. However, East Timor veterans had higher counts of stressors on each of the ‘separation’, ‘different culture’, ‘other people’ and ‘work frustration’ factors. This suggests that a more warlike deployment may be associated with greater general frustration with the more routine aspects of life on deployment.

Previous research has shown that events such as witnessing atrocities or massacres [32] is associated with worse mental health, after accounting for the effect of combat on deployment. Likewise seeing [2] or handling and recovering of war dead [33] were associated with worse mental health outcomes and symptoms of PTSD. In the groups deployed to Bougainville and East Timor, the scale measuring seven objective stressors included similar exposures to dead bodies, deaths and injuries of colleagues and witnessing human degradation, but these were not associated with worse health outcomes once other types of stressors were accounted for. One possibility is that those deployed may have been “inoculated” against worse health outcomes due to repeated exposures to these types of events in these and previous deployments [34]. Information on other objective stressors, such as being shot at and being wounded or injured that have been shown to be associated to worse mental health outcomes, [15] was not collected in this study, and if such items we included we may have been more likely to reveal an association between objective exposures and worse mental health outcomes.

In contrast a scale of subjective traumatic stressors which included fear of injury or death and a belief that one’s action resulted in injury of death of another, was associated with PTSD and increased reporting of physical and psychological symptoms. This finding is consistent with work showing the subjective exposure of perceived threat to life is one of the strongest predictors of PTSD [2,16-18] and other research that showed those who believe their actions resulted in someone’s death had worse mental health outcomes [3]. Despite the associations between subjective traumatic stressful experiences and both PTSD and physical and psychological symptoms, both the Bougainville and East Timor veterans reported that they were less affected in 2008 by stressful events that occurred while they were on deployment Most participants stated they were no longer affected ‘at all’ by the events listed in the TSES-R2 scale. This is consistent with previous research which has shown that there are many enduring effects of traumatic events that the participants in the studies do not apparently register [35].

As well as the subjective traumatic stressors some non-traumatic deployment stressors were shown to be associated which each of the health outcomes considered. Non-traumatic deployment stressors, unrelated to combat, have previously been shown to be a strong predictor of PTSD in US Marines returning from deployments in the MEAO [1]. In our study stressors concerning ‘work frustration’ were most frequently associated with worse health outcomes while other types of non-traumatic stressors were also associated with worse health outcomes. While many of the traumatic stressors are likely to be caused by unpredictable events, the affect of some of the non-traumatic deployment stressors (such as boredom, sorting out problems and separation) may be able to be modified by the ADF through training or strategies to reduce the level of these more general worries on deployments and subsequently help improve wellbeing of returning soldiers [1].

### Strengths and weaknesses of the study

The similar design and questionnaire items used in the Bougainville and East Timor studies have allowed comparison of health outcomes resulting from deployments that included both warlike operations and peacekeeping deployments. A strength of the studies was that the data were collected simultaneously, and the operations occurred over similar time periods (1997–2003 and 1999–2005 respectively). In addition the geographic locations of the deployments relative to Australia were similar, yet the traumatic exposure levels and non-traumatic stressors differed.

Nevertheless, the results presented should be interpreted with some caveats. Because of the increased frequency of overseas deployments within the ADF it is difficult to attribute a participant’s health status in 2008 to the stressors experienced on earlier deployments to Bougainville and East Timor. It may be that those ADF members exposed to traumatic events and non-traumatic stressors on one deployment are more likely to experience similar stressors on subsequent deployments (for example to the Middle East) due to their specific role within the ADF. Nevertheless, adjustment for previous deployments to the Middle East had little effect on the results presented.

The cross-sectional design of the studies meant that information on exposure on deployment (traumatic experiences and non-traumatic stressors) and health outcomes was collected at the same time. It is possible that people who were currently experiencing health problems were more likely to associate these with stressful deployment events [36]. Additionally, the median time between the first deployment and questionnaire completion was eight years both Bougainville and East Timor participants, so further recall bias is possible.

The response rates among Bougainville and East Timor veterans were 49 % and 46 % respectively and ex-serving personnel were particularly under-represented. This may have resulted in underestimation of the prevalence of health symptoms and mental health conditions. The lower completion rates of the TSES-R scale were due in part to the questions being placed towards the end of the questionnaire and the requirement that each of the individual items was completed in order to calculate the overall score. However, those who did not complete this section had a similar demographic profile to those who did (Table 2).

## Conclusion

In an era where there is a focus on Defence personnel being exposed to combat and casualties in locations such as Iraq and Afghanistan, the traumatic events and non-traumatic operational stressors and health outcomes experienced by peacekeepers have received less attention. Despite differences in frequency of traumatic events and non-traumatic stressors reported on both non-warlike and warlike deployments, in both studies, those who reported more subjective traumatic exposure and different types of deployment stressors were more likely to report poorer physical and mental health outcomes more than eight years after deployment. Strategies to better prepare, identify and treat those exposed to both traumatic events and other operational stressors on deployment should be considered for military personnel deployed on both warlike and peacekeeping operations.

## Competing interests

The authors declare that they have no competing interests.

## Author’s contributions

MW carried out the statistical analysis and drafted the manuscript. All other authors contributed text to the manuscript, made suggested of other analyses to include and helped interpret the results. All authors read and approved the final manuscript.

## Funding

This work was funded as part of the Australian Near North Deployment Health Surveillance Program (DHSP), by the Department of Defence and the Department of Veterans Affairs. All authors were researchers or Chief investigators on the DHSP studies.

## Pre-publication history

The pre-publication history for this paper can be accessed here:

http://www.biomedcentral.com/1471-244X/12/88/prepub

## References

[B1] Booth-Kewley S Correlates of posttraumatic stress disorder symptoms in Marines back from war J Trauma Stress 2010 23 1 69 77 20104587 10.1002/jts.20485

[B2] Phillips CJ Risk factors for posttraumatic stress disorder among deployed US male marines BMC Psychiatry 2010 10 52 10.1186/1471-244X-10-52 20579379 PMC2912797

[B3] Maguen S The impact of reported direct and indirect killing on mental health symptoms in Iraq war veterans J Trauma Stress 2010 23 1 86 90 20104592 10.1002/jts.20434

[B4] Dohrenwend BP The psychological risks of Vietnam for U.S. veterans: a revisit with new data and methods Science 2006 313 5789 979 982 10.1126/science.1128944 16917066 PMC1584215

[B5] Sareen J Is peacekeeping peaceful? A systematic review Can J Psychiatry 2010 55 7 464 472 20704774 10.1177/070674371005500710

[B6] Ward W Psychiatric morbidity in Australian veterans of the United Nations peacekeeping force in Somalia Aust N Z J Psychiatry 1997 31 2 184 193 10.3109/00048679709073819 9140624

[B7] MacDonald C Mental health, physical health, and stressors reported by New Zealand Defence Force peacekeepers: a longitudinal study Mil Med 1998 163 7 477 481 9695614

[B8] Hotopf M The health effects of peacekeeping (Bosnia, 1992–1996): a cross-sectional study–comparison with nondeployed military personnel Mil Med 2003 168 5 408 413 12775179

[B9] Maguen S The stressors and demands of peacekeeping in Kosovo: predictors of mental health response Mil Med 2004 169 3 198 206 15080239 10.7205/milmed.169.3.198

[B10] Michel PO Lundin T Larsson G Stress reactions among Swedish peacekeeping soldiers serving in Bosnia: a longitudinal study J Trauma Stress 2003 16 6 589 593 10.1023/B:JOTS.0000004084.85732.86 14690357

[B11] Han CS Kim YK Psychiatric symptoms reported by international peacekeeping personnel in the Western Sahara Desert J Nerv Ment Dis 2001 189 12 858 860 10.1097/00005053-200112000-00008 11794580

[B12] Thomas S Serving in Bosnia made me appreciate living in Bristol: stressful experiences, attitudes, and psychological needs of members of the United Kingdom Armed Forces Mil Med 2006 171 5 376 380 16761885 10.7205/milmed.171.5.376

[B13] Hotopf M The health effects of peace-keeping in the UK Armed Forces: Bosnia 1992–1996. Predictors of psychological symptoms Psychol Med 2003 33 1 155 162 12537046 10.1017/s0033291702006840

[B14] Orsillo SM Psychiatric symptomatology associated with contemporary peacekeeping: an examination of post-mission functioning among peacekeepers in Somalia J Trauma Stress 1998 11 4 611 625 10.1023/A:1024481030025 9870217

[B15] Hoge CW Combat duty in Iraq and Afghanistan, mental health problems, and barriers to care N Engl J Med 2004 351 1 13 22 10.1056/NEJMoa040603 15229303

[B16] Kolkow TT Post-traumatic stress disorder and depression in health care providers returning from deployment to Iraq and Afghanistan Mil Med 2007 172 5 451 455 17521088 10.7205/milmed.172.5.451

[B17] Vogt DS Tanner LR Risk and resilience factors for posttraumatic stress symptomatology in Gulf War I veterans J Trauma Stress 2007 20 1 27 38 10.1002/jts.20187 17345645

[B18] Iversen AC Risk factors for post-traumatic stress disorder among UK Armed Forces personnel Psychol Med 2008 38 4 511 522 18226287 10.1017/S0033291708002778 PMC3785135

[B19] McFarlane AC Bryant RA Post-traumatic stress disorder in occupational settings: anticipating and managing the risk Occup Med (Lond) 2007 57 6 404 410 10.1093/occmed/kqm070 17728313

[B20] ADF Pay and Conditions Manual Chapter 17 2010, cited 2010 21/07/2010

[B21] Swann J Hodson S A psychometric analysis of the traumatic stress exposure scale - revised Research report 09/04 2004 Department of Defence, Canberra 39

[B22] Steele N Development of the Mental Health Screen: A history. PRTG Technical Brief 04/05 2005 Department of Defence, Canberra

[B23] Kelsall HL Symptoms and medical conditions in Australian veterans of the 1991 Gulf War: relation to immunisations and other Gulf War exposures Occup Environ Med 2004 61 12 1006 1013 10.1136/oem.2003.009258 15550607 PMC1740679

[B24] Unwin C Health of UK servicemen who served in Persian Gulf War Lancet 1999 353 9148 169 178 10.1016/S0140-6736(98)11338-7 9923871

[B25] Andrews G Slade T Interpreting scores on the Kessler Psychological Distress Scale (K10) Aust N Z J Public Health 2001 25 6 494 497 10.1111/j.1467-842X.2001.tb00310.x 11824981

[B26] Weathers FW Litz BT Herman DS Huska JA Keane TM The PTSD checklist (PCL): Reliability, validity, and diagnostic utility 1993 Paper presented at the 9th Annual Meeting of the International Society for Traumatic Stress Studies, San Antonio, TX

[B27] American Psychiatric Association Diagnostic and Statistical Manusal of Mental Disorders 1994 4 APA, Washington

[B28] Forbes D Creamer M Biddle D The validity of the PTSD checklist as a measure of symptomatic change in combat-related PTSD Behavior Research and Therapy 2001 39 977 986 10.1016/S0005-7967(00)00084-X 11480838

[B29] Blanchard EB Psychometric properties of the PTSD Checklist (PCL) Behav Res Ther 1996 34 8 669 673 10.1016/0005-7967(96)00033-2 8870294

[B30] SAS Institute Inc 2002 SAS Institute Inc, Cary, NC SAS(r) software version 9.2

[B31] StataCorp Stata Statistical Software: Release 10 2007 StataCorp LP, College Station

[B32] Sareen J Combat and peacekeeping operations in relation to prevalence of mental disorders and perceived need for mental health care: findings from a large representative sample of military personnel Arch Gen Psychiatry 2007 64 7 843 852 10.1001/archpsyc.64.7.843 17606818

[B33] McCarroll JE Ursano RJ Fullerton CS Symptoms of posttraumatic stress disorder following recovery of war dead Am J Psychiatry 1993 150 12 1875 1877 8238646 10.1176/ajp.150.12.1875

[B34] Meichenbaum D Novaco R Stress inoculation: a preventative approach Issues Ment Health Nurs 1985 7 1–4 419 435 3854020 10.3109/01612848509009464

[B35] Smid GE Delayed posttraumatic stress disorder: systematic review, meta-analysis, and meta-regression analysis of prospective studies J Clin Psychiatry 2009 70 11 1572 1582 10.4088/JCP.08r04484 19607763

[B36] Wessely S Stability of recall of military hazards over time. Evidence from the Persian Gulf War of 1991 Br J Psychiatry 2003 183 314 322 10.1192/bjp.183.4.314 14519609

